# Magnetically triggered drug release from nanoparticles and its applications in anti-tumor treatment

**DOI:** 10.1080/10717544.2016.1256001

**Published:** 2017-02-09

**Authors:** Xin Hua, Qin Yang, Zhimin Dong, Jiashuo Zhang, Wanjiang Zhang, Qiudong Wang, Shengnan Tan, Hugh D. C. Smyth

**Affiliations:** 1 Division of Bacterial Diseases, State Key Laboratory of Veterinary Biotechnology, Harbin Veterinary Research Institute, Chinese Academy of Agricultural Sciences, Harbin, China,; 2 Tianjin Animal Science and Veterinary Research Institute, Tianjin, China,; 3 College of Wildlife Resources, College of Life Science, Northeast Forestry University, Harbin, PR China, and; 4 College of Pharmacy, The University of Texas at Austin, Austin, TX, USA

**Keywords:** Magnetic nanoparticles, oscillating magnetic field, controlled release, doxorubicin

## Abstract

The objective of this study was to describe the magnetic nanoparticle–drug conjugates for improved control of drug delivery and drug release. The widely used anticancer agent Doxorubicin (DOX) was successfully conjugated *via* amine groups to the carboxylic functional groups coating magnetic nanoparticles (fluidMAG-CMX). Following purification of the nanoparticles, the conjugation of DOX on fluidMAG-CMX was confirmed using FTIR spectroscopy and confocal microscopy. The observed drug loading capacity of DOX was 22.3%. Studies of magnetically triggered release were performed under an oscillating magnetic field (OMF). DOX exhibited a significant release percentage of 70% under an OMF, as compared with the release in enzyme. A magnetic field turn-on and turn-off experiment was also conducted to confirm the control of drug release using this triggered system. *In vivo* experiments indicated that the tumor-inhibitory rate of CMX–DOX NPs under a magnetic field was higher than the other control groups. According to the toxicity assessments, CMX–DOX NPs were not noticeably toxic to mice at our tested dose.

## Introduction

In recent years, a large volume of research has focused on proposing new strategies to fabricate drug nanocarriers, for example, dendrimers (Yellepeddi & Ghandehari, 2016), nanoparticles (Beiranvand et al., 2016; DeMarino et al., 2016; Chen et al., 2016) and nanogels (Soni & Yadav, 2016). Although these nanocarriers can improve the permeability and retention of drugs due to their small size (Davis et al., 2008; Farokhzad & Langer, 2009; He et al., 2010), these delivery systems are not specific and cannot control drug release accurately. Compared with other promising nanocarriers, magnetic nanoparticles (MNPs), commonly referred to as superparamagnetic iron oxide nanoparticles (SPIONs), exhibit certain special advantages for hyperthermia and targeting because of their intrinsic magnetic property. Nanoparticles generate heat when exposed to a high-frequency magnetic field (Brule et al., 2011), and this heat can be applied for localized hyperthermia cancer therapy (Liu et al., 2011). When manipulated by an external magnetic field, drugs combined with magnetic nanoparticles can be delivered to targeted sites, thereby avoiding side effects. These features lend promise to potential applications of magnetic nanoparticles in biomedicine (Long et al., 2015; Bao et al., 2016; Mohammed et al., 2016). Hence, much effort has been devoted to the development of targeted delivery and release in a controlled manner *via* hyperthermia in a local high-frequency alternating magnetic field (HAMF) (Liu et al., 2008; Satarkar & Hilt, 2008; Oliveira et al., 2013). Satarkar & Hilt (2008) reported the use of a HAMF to trigger on-demand pulsatile drug release from magnetic hydrogel nanocomposites. Oliveira et al. used polymersome-encapsulated doxorubicin together with SPIONs for targeted drug release under a HAMF^15^ (14 mT at 750 kHz). However, in clinical trials in Germany, the field strength was limited to the range of 3–14 kA/m (at a frequency of 100 kHz) in order to limit patient discomfort, which depended on the region treated (Jordan et al., 2006). Previously, we reported the ability of magnetic nanoparticles to cause the disruption of a biopolymer under an oscillating magnetic field (OMF) (McGill et al., 2009). In this report, we continued to investigate the drug release profile from magnetic nanoparticles under a low-frequency OMF.

Covalent binding of drugs to magnetic nanoparticles results in the formation of stable chemical structures, and these drugs can be released under specific conditions, such as in the presence of proteases (Jeffrey et al., 2006). In this study, we used simple magnetic nanoparticles as a model nanocarrier, to conjugate with different types of drugs *via* a peptide bond. Due to the different chemical structures of drugs, they had different conjugation capacities and drug release profiles. There have been similar works previously, which reported increased cytotoxicity to cancer cells (Chen et al., 2008; Zhao et al., 2012). All of these reports have shown that drugs can only be released in low-pH conditions and need the presence of lysozymes, which are typical conditions in tumor cells. This suggests that this type of nanocarrier can be used in a specific anti-tumor model. In this study, we hypothesize that a magnetic field can be used to trigger drug release, expanding the potential applications of the drug delivery model. To the best of our knowledge, there have been few reports on triggered drug release from drug–MNP conjugates under a low-frequency OMF.

## Materials and methods

### Materials

Doxorubicin hydrochlorate was obtained from Chemie Tek. NHS-sulfo and 1-ethyl-3-[3-dimethylaminopropyl] carbodiimide hydrochloride (EDC) were purchased from Thermo Scientific (Waltham, MA). fluidMAG-CMX (polymer matrix was carboxymethyldextran) was obtained from Chemicell Company (Berlin, Germany). MACS Separation 20 μL columns were purchased from Miltenyi Biotec (Auburn, CA). All other chemicals were HPLC grade.

### Synthesis of drug conjugated nanoparticles

An EDC crosslinker and sulfo-NHS were used to conjugate drugs to MNPs. Briefly, 12.5 mg EDC and 13 mg sulfo-NHS were dissolved in 1 mL 0.5 M MES buffer (pH 9.5). This solution was then mixed with 1 mL fluidMAG-CMX (25 mg/mL), and allowed to react for 2 h in the dark. The mixtures were centrifuged at 15,000 rpm for 5 min and separated, and the residuals were washed 3 times with a 0.1 M MES buffer to remove excess EDC and sulfo-NHS. The mixture was suspended in a solution of DOX of the same molecular weight, and incubated for 6 h at room temperature in the dark. The conjugates were separated and washed until colorless with DI water, and the dried samples were stored at 4 °C. All of the supernatants were collected and separated using a μ-column (MACS Separation 20 μ columns contain an optimized matrix to generate a strong magnetic field when placed in a permanent magnet) to eliminate residual MNPs for indirect calculation of the drug loading content.

### Characterization of drug–CMX conjugates

To confirm the formation of drug–CMX conjugates, a Nicolet IR 100 Fourier transform infrared (FTIR) spectrometer (Thermo Electron Corporation) and Confocal Leica TCS SP5 II Fixed Stage (Upright) Microscope (Leica Microsystems Inc.) were used. 10 μL of fluidMAG-CMX was dropped on a formvar film on 300 square mesh copper grids and dried for TEM imaging using a Tecnai™ transmission electron microscope (FEI Company). The particle size distribution was characterized by dynamic light scattering (DLS) (Zetasizer Nano ZS, Malvern, UK).

### Drug loading content

Considering the UV absorption interference and fluorescence quenching effects of magnetic nanoparticles, the drug loading capacity was indirectly calculated as follows:

$$\mathrm{Drug}\;\mathrm{loading}\;\mathrm{efficiency}\;(\%)=\frac{100\times (W_{\mathrm{feed}\;\mathrm{drug}}-W_{\mathrm{free}\;\mathrm{drug}})}{W_{\mathrm{feed}\;\mathrm{drug}}}$$

To determine the DOX loading efficiency, we utilized an Infinite M2000 TECAN UV–Vis spectrometer (Tecan Infinite M200, Switzerland) at a wavelength of 485 nm.

### Analysis of drug release from magnetic nanoparticles

Crude protease from a bovine pancreas (Sigma-Aldrich Co.) was used to simulate intracellular lysosomal conditions. The conjugates were suspended in 1 mL of PBS buffer containing 0.1 mg/mL crude protease. The release was performed in a constant-temperature shaker at 37 °C. MNP suspensions were centrifuged at 10,000 rpm to separate the cleaved drug from the MNPs at regular time intervals. To ensure that there were no residual MNPs, free drugs were passed through μ-columns. The samples were collected for measurement. Normal release without crude protease was performed under the same conditions as the control.

For the study of triggered release, we utilized an oscillating magnetic field (OMF) as an external field. 1 mL of the CMX–drug conjugate was placed in an OMF apparatus (NanoTherics Ltd., UK) for exposure over 1–10 h. The nominal frequency was 100 kHz, with an amplitude of 0.56 kA/m. After being centrifuged at 10,000 rpm for 5 min, the supernatants were collected and passed through MACS Separation 20 μ columns for purification. All experiments were performed in triplicate. The determination of drug release was performed by a UV spectrophotometer.

### *In vitro* cellular toxicity

The cytotoxicity of CMX–DOX was determined using MTT assay. Hela cells were plated in 96-well culture plates and incubated for 24 h at 37 °C. Then cells were exposed to positive control group of free DOX for 24 h. Another two group were exposed to CMX–DOX in the presence and absence of OMF. MTT, which dissolved in phosphate-buffered saline at a dose of 2 mg/mL, was added. After incubation for 4 h at 37 °C, the purple formazan crystals were dissolved with 100 ll dimethyl sulfoxide and the absorbance was measured at 570 nm in an ELISA reader (Thermo Molecular Devices Co., Union City). IC50 value was calculated according to the linear regression of cell viability ratio. The equation of cell viability was as following:

$$\mathrm{Cell}\;\mathrm{viability}\;\mathrm{ratio}\;\left(\%\right)={\mathrm{OD}}_{\mathrm{treated}}/{\mathrm{OD}}_{\mathrm{control}}\times 100\%$$

### *In vivo* anti-tumor efficiency

BALB/c mice were divided into seven groups with 10 mice in each group. Next, 2 × 10^6^ (HeLa cells) were injected into the backs of mice for tumor formation. All the mice were anesthetized with isoflurane before drug injection when the tumor reached a size of 80 mm^3^. As a negative control, mice injected with or without PBS were labeled as groups 1 and 2. In groups 3, 4 and 5, the mice were treated with CMX (same amount as group 5), DOX (10 mg/kg), and CMX–DOX (10 mg/mL free DOX equivalent), respectively. Groups 6 and 7 were injected with CMX (same amount as group 5) and CMX–DOX (10 mg/mL free DOX equivalent), respectively, and followed by OMF treatment for 6 h and OMF treatment at intervals, respectively. In the next 21 days, the tumor sizes were measured by a caliper at regular intervals and calculated according to the formula, Volume ¼ (Tumor Length) * (Tumor Width)^2^/2. All experimental work involving animals was performed according to the guidelines recommended by the animal welfare and ethics of the Heilongjiang Animal Ethics Committee at the Heilongjiang science and technology government agency (Harbin, People’s Republic of China) and was approved and supervised by the commissioner for animal welfare at the Harbin Veterinary Research Institute (HVRI) representing the Institutional Animal Care and Use Committee.

### Toxicity

For toxicity experiments, blood samples were collected every 24 h after the injection of different drugs for the blood biochemistry assay, and the mice were later sacrificed. The serum chemistry data and complete blood panel were measured. For histology analysis, the harvested tumor of mice in control group and in CMX–DOX under OMF treated group were fixed with 10% neutral buffered formalin, processed into paraffin, sectioned at a thickness of 8 microns, stained with H&E, and examined by a digital microscope (Leica QWin) (Table 1).

**Table 1.** Biochemical parameters for the control group and CMX–DOX under the OMF group.

		CMX–DOX injected (20 mg/kg)		
	Control	1 day	7 days	30 days
ALT (IU)	54.6 ± 6.7	50.6 ± 4.9	55.1 ± 3.9	57.5 ± 7.3
AST (IU)	144.2 ± 9.2	159.4 ± 8.8	155.0 ± 10.6	149.3 ± 6.1
ALP (IU)	189.6 ± 17.2	188.0 ± 9.8	182.1 ± 12.6	184.6 ± 15.9
BUN (mmol)	19.7 ± 2.9	18.7 ± 2.6	20.0 ± 3.5	21.6 ± 3.6
WBC (10^9^/L)	10.6 ± 2.3	11.5 ± 2.1	10.8 ± 1.9	11.2 ± 1.1
RBC (10^12^/L)	9.8 ± 1.8	9.6 ± 2.1	8.8 ± 0.98	10.5 ± 1.1
HCT (%L/L)	0.56 ± 0.10	0.60 ± 0.08	0.62 ± 0.06	0.54 ± 0.05
HGB (g/L)	16.7 ± 2.1	15.5 ± 2.3	14.9 ± 2.6	16.5 ± 1.9
MCV (fL)	58.6 ± 5.3	55.9 ± 4.8	60.1 ± 8.6	59.3 ± 6.1
MCHC (g/mL)	22.0 ± 3.3	21.1 ± 4.5	23.4 ± 1.8	23.2 ± 2.5
MPV (10^12^/L)	6.0 ± 0.92	5.4 ± 0.51	5.7 ± 0.85	6.1 ± 0.64
PLT (k/mL)	988.0 ± 125.2	849.5 ± 99.5	1062.8 ± 153.9	965.2 ± 123.0

## Results and discussion

### Characterization of fluidMAG-CMX

**Figure 1.**
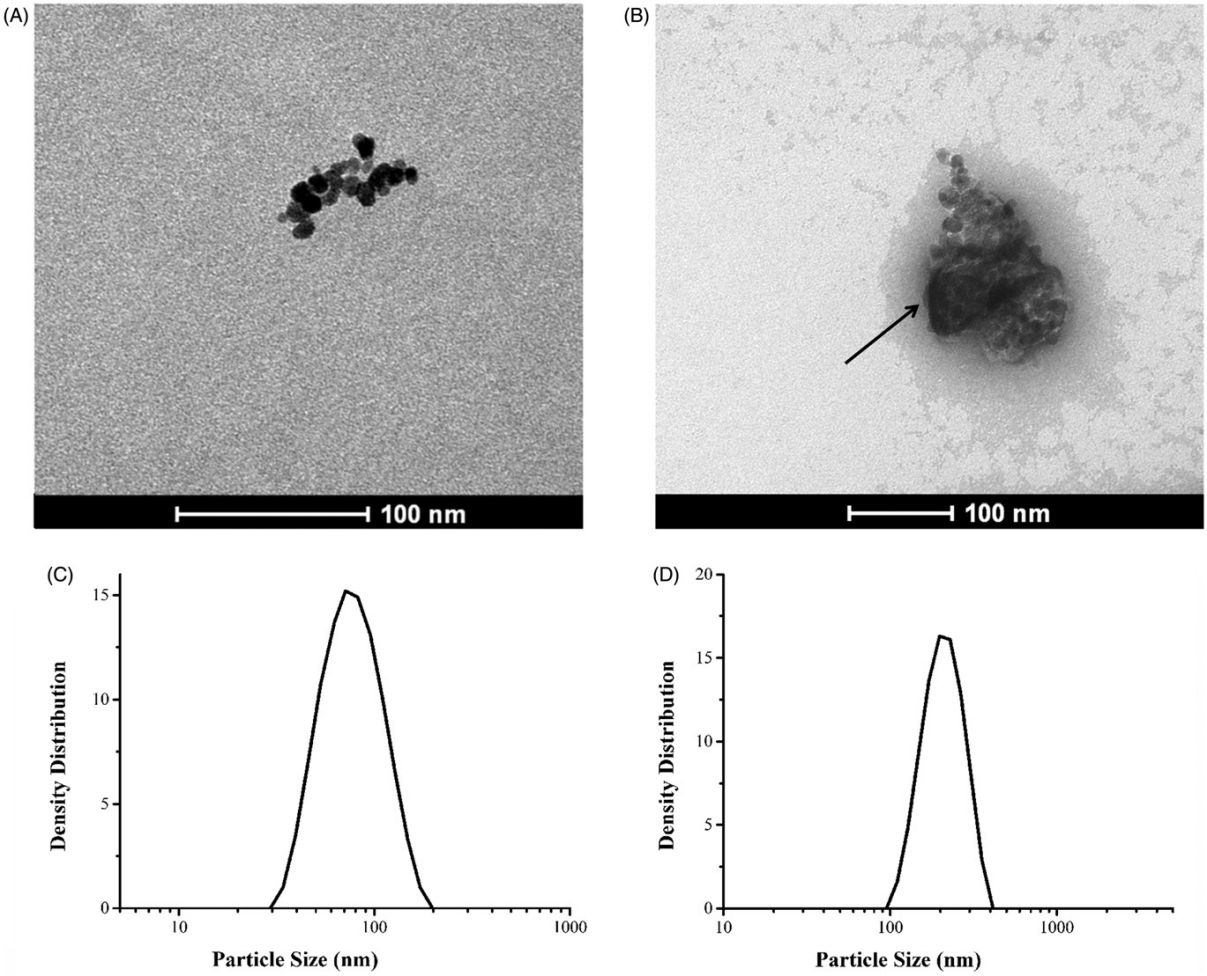
TEM image of (A) fluidMAG-CMX particles; (B) fluidMAG-CMX with positive staining by uranyl acetate. Particle size distribution by DLS of (C) fluidMAG-CMX; (D) CMX–DOX.

Uranyl acetate was used as a positive contrast in TEM observations, for clear identification of the polymer matrix (carboxymethyldextran) of fluidMAG-CMX, as shown in Figure 1(B). The arrow indicates the magnetic core of fluidMAG-CMX, while the substance around the core was carboxymethyldextran, with uranyl acetate as the positive contrast. The image analysis software Image J was utilized to measure the particle size. The mean diameters of fluidMAG-CMX and its magnetic core were 115 nm and 61 nm, respectively. This is consistent with the results of DLS, which showed particle diameters of 34–171 nm.

### Characterization and quantification of drug–CMX conjugates

**Figure 2.**
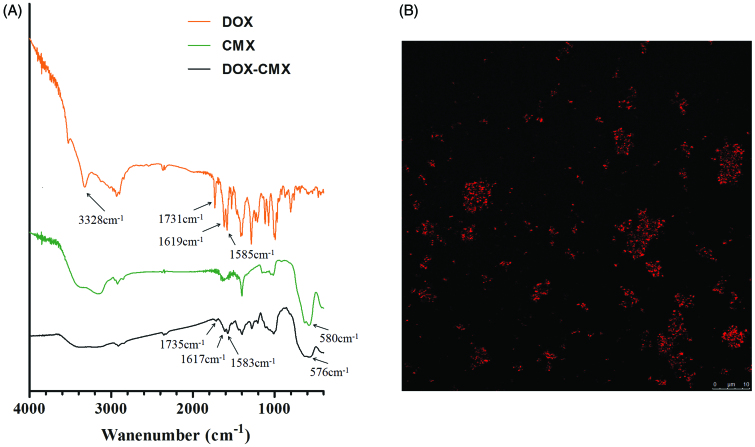
(A) FTIR spectra of DOX, CMX, and CMX–DOX. (B) Confocal microscopic image of CMX–DOX.

Because DOX exhibits strong fluorescence, confocal microscopy was used to confirm the success of covalent binding of DOX with CMX under the experimental conditions. The emission wavelength was 495 nm and the excitation wavelength was 547 nm. The red spots in Figure 2(B) are CMX–DOX conjugates.

FTIR spectroscopy was utilized to further confirm the formation of DOX–CMX conjugates, as shown in Figure 2(A). Standard DOX shows the characteristic IR absorption band at 1731 cm^−1^ due to the stretching vibration of the carbonyl group at the 13-keto position. This band shifted to 1735 cm^−1^ after conjugation. Additionally, the bands at 1619 and 1585 cm^−1^, attributed to the stretching vibrations of the two carbonyl groups of the anthracene ring, were shifted to 1617 and 1583 cm^−1^, respectively. In addition, the broad band near 3328 cm^−1^ refers to the vibration of the –NH_2_ group. After the DOX is conjugated to fluidMAG-CMX, the wide broad band near 3328 cm^−1^ disappears and a new peak appears at 576 cm^−1^ in Figure 2(A), indicating the presence of magnetic nanoparticles.

These results demonstrate the successful conjugation of DOX to the magnetic nanoparticles *via* an amido linkage, demonstrating that fluidMAG-CMX nanoparticles with carboxyl groups (–COOH) are convenient for further conjugation with different drugs containing amino groups.

As shown in the results in Figure 1(D), the CMX–DOX particles spanned a narrow range of diameters, ranging from 117 to 229 nm, with PDI of 0.279. This finding indicates that drug conjugation did not induce any aggregation of magnetic nanoparticles.

The drug loading capacity was indirectly calculated using the unbound drug as the equation. The estimated loading efficiency of DOX was 22.3%. The amount of DOX grafted per milligram of fluidMAG-CMX nanoparticles was 59 μg.

### Release study

The mechanisms of DOX release from the conjugates inside target cells were similar. Once the DOX–CMX conjugates are brought into the cell, lysosomes containing proteases clear these exogenous materials. These proteases are capable of hydrolyzing the amide bond between DOX and CMX, releasing free drugs inside the target cell and entering the cellular cytosol. Herein, we simulated the intracellular lysosomal conditions by using a PBS buffer containing 0.1 mg/mL crude protease, to investigate the release profiles of different drugs.

**Figure 3.**
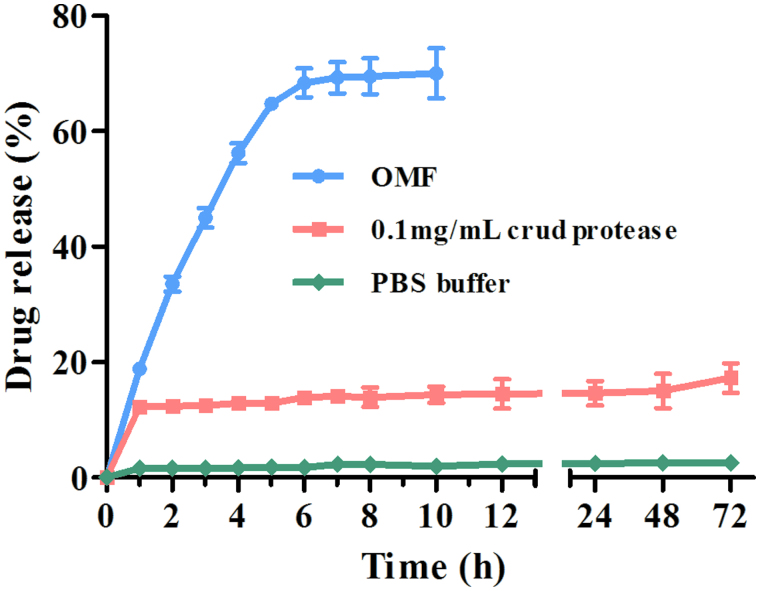
*In vitro* drug release under different conditions (PBS group, crude protease group, and OMF group).

As shown in Figure 3, there is a burst release of DOX in the first hour, in which a total of 12.3% of drug is released from the CMX–DOX conjugates with a release rate of 27.5 μg/h. Then, the release rate decreases to 0.2 μg/h in the next 71 h. Only 17.3% of the total DOX is released in 72 h. This release profile is consistent with previous reports (Chen et al., 2008). In the control group of normal release (without crude protease), there is no insignificant passive release of DOX, and the drug release percentage is less than 3%.

As introduced above, MNPs can be externally manipulated in several different ways. In these experiments, we wanted to test the hypothesis that MNPs conjugated with drugs could be used to trigger drug release or to increase release rates using an external oscillating magnetic field.

From Figure 3, we can see that DOX shows a linearly increased trigger release under an OMF in the initial 6 h, with a mean release rate of 113.3 μg/h. There is no significant increase of these two drugs from 7 to 10 h. In comparison, a significant increase of drug release is observed under an OMF, with which 70% of the total DOX is released in just short of 10 h. This shows that magnetically triggered release results in a 4-fold increase compared with DOX release in the crude protease.

**Figure 4.**
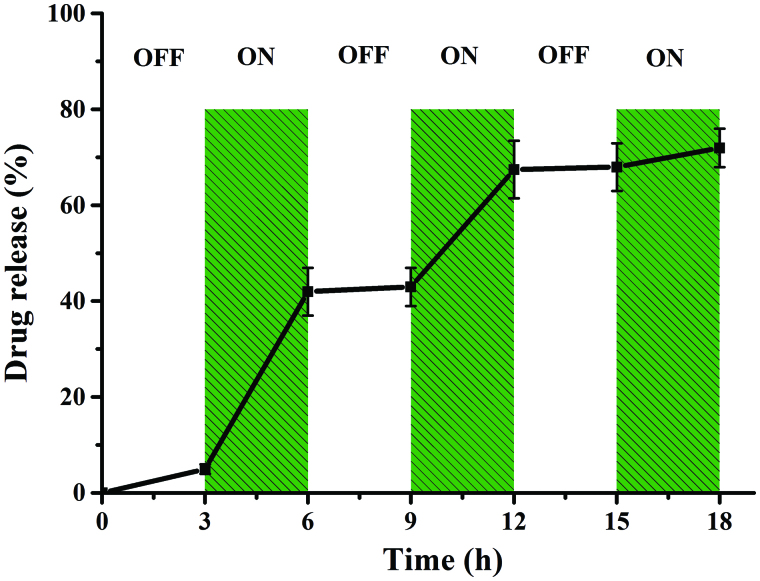
Switched turn-off and turn-on release of CMX–DOX under an OMF.

To investigate this controllable release property, we designed a turn-on and turn-off switch experiment (Figure 4). An OMF was applied to the particles from 0 to 18 h, turned off from 0 to 3 h, switched on at 36 h, and so on until 18 h was reached. It can be observed that during the “off” time periods, low or no drug release is observed. The drug release during the first “off” period is attributed to residual drug. A significant increase in drug release is observed during the “on” time periods. In particular, in the first “on” period of DOX release, 37% of the total DOX is released at a rate of 123.3 μg/h, which is higher than other “on” periods. Approximately 68.5% of the total DOX is released at the end of the second “on” period, which is consistent with the result of sustained release under an OMF. In the third “ON” period, only 5% free DOX is released. Collectively, these studies show the potential of magnetic nanoparticles as a controllable release platform for various drugs.

**Figure 5:**
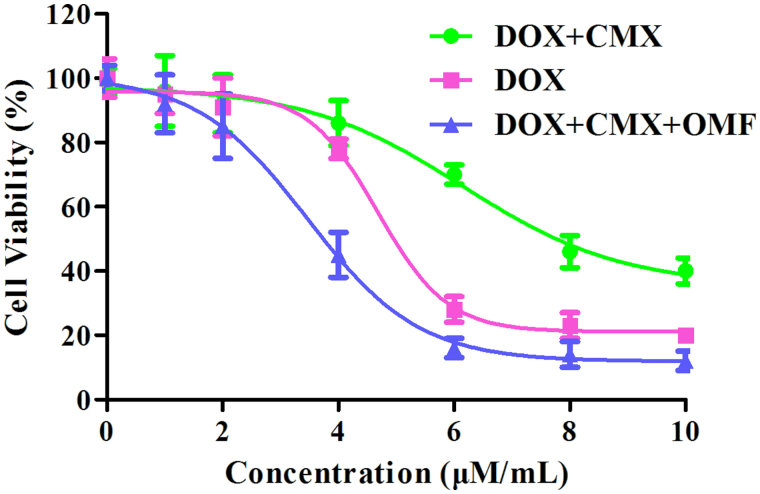
Cell viability of Hela cells in the absence or presence of OMF after treatment with CMX–DOX NPs by MTT assay, DOX as a positive control.

MTT assay were conducted to investigate the toxicity of CMX–DOX toward Hela cell *in vitro*. In Figure 5, the IC_50_ of CMX–DOX, DOX and CMX–DOX under OMF were 6.15 μM, 4.68 μM and 3.46 μM, respectively.

**Figure 6.**
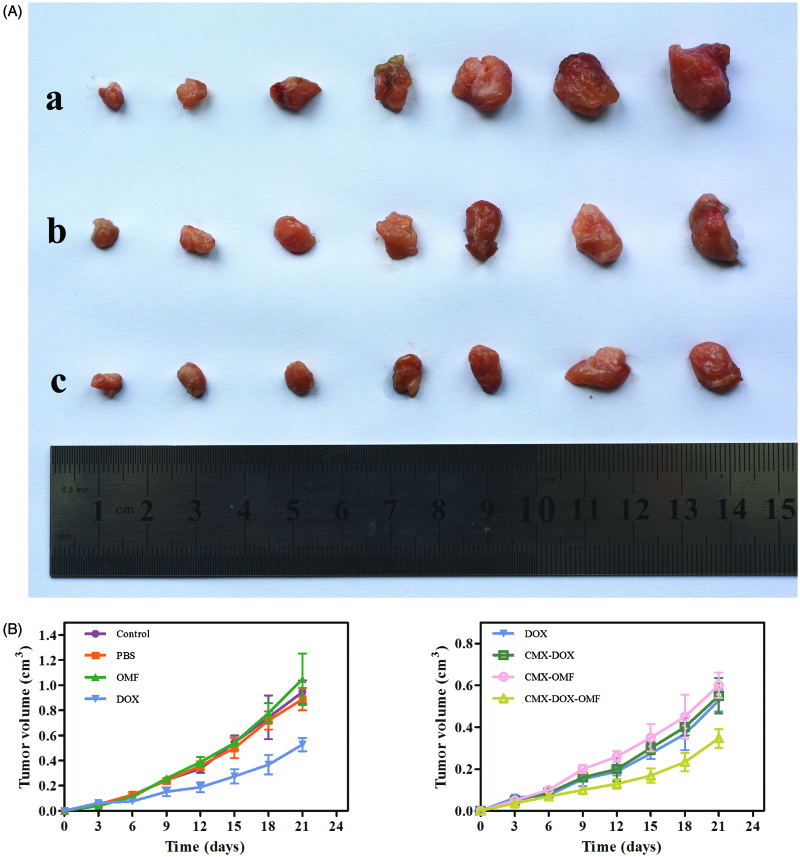
Growth of tumor after various treatments over 21 days. (A) Representative photos of tumors after various treatments, taken 21 days after treatment. a. control group, b. free DOX and c. CMX–DOX under OMF. (B) The average changes in tumor volume over 21 days after different treatments, including the PBS group, CMX without OMF group, free DOX group, CMX–DOX without OMF group, CMX–OMF group, CMX–DOX–OMF group, as well as control group.

We designed animal experiments to demonstrate the *in vivo* therapeutic effect. Seven groups of balb/c mice at 10 mice per group were used in our experiment. Identical amounts of saline, DOX and CMX–DOX were injected by a single intratumoral injection. In groups 6 and 7 with CMX and CMX–DOX injections, the OMF was applied for 6 h. Over the next 21 days, the tumor size was monitored every three days. Figure 6(B) shows the tumor growth in each group. In the control groups, the tumor growth is unaffected by treating with PBS and MNPs. Compared with the control groups, the mice treated with free DOX showed moderate growth inhibition. The MNP–DOX group showed a lower growth inhibition than the DOX group, indicating that there was little release in the tumor site. This is consistent with the results of release *in vitro*. The mice in group 7, applied with an OMF after MNP–DOX injection, showed the smallest tumor size. The mean tumor size in this group was only 0.23 cm after 21 days. This result indicates that the application of an OMF led faster release of DOX at the tumor site, compared with the no-OMF treatment group, and showed the highest efficacy in tumor reduction.

**Figure 7.**
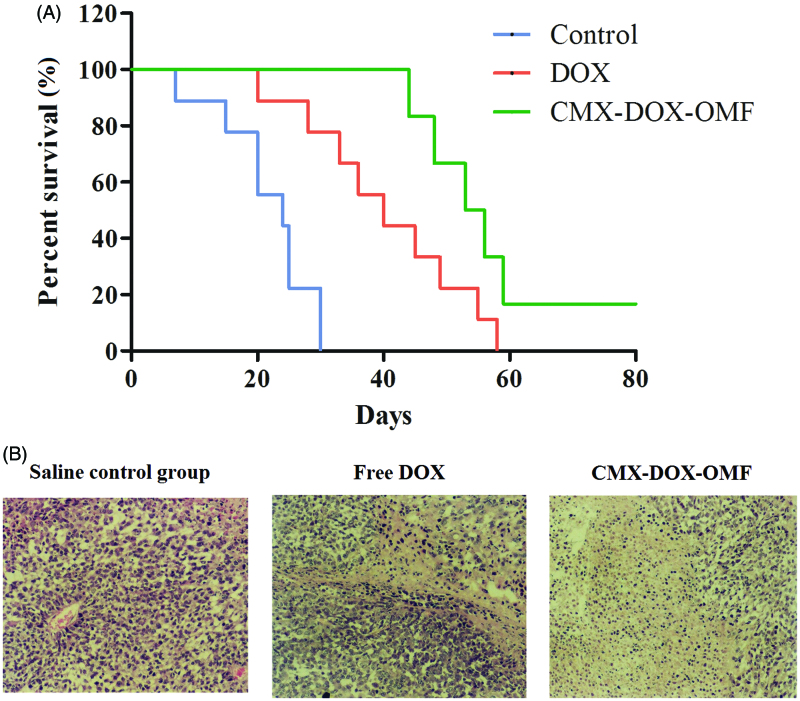
(A) Survival curves of various groups of mice after different treatments. (B) Detection of apoptosis with hematoxylin and eosin (H&E) staining in tumor tissues (×100).

*In vivo* toxicity of DOX and prepared formulations was assessed by the systematic hematological assessments and histopathology investigations of different organs. According to the previous reports, free DOX toxicity induced by the generation of radicals was reported to cause hepatic and cardiac toxicity (Injac & Strukej, 2008; Iqbal et al., 2008; Bulucu et al., 2009). Herein, there is no need to verify the toxicity of DOX. Compared with the control group, all measured parameters of the CMX–DOX-injected group were within normal ranges. In particular, the liver function indicators, alanine aminotransferase (ALT), alkaline phosphatase (ALP) and aspartate aminotransferase (AST), all exhibited normal levels. H&E staining technique was applied to observe the morphology of the apoptotic cells in tumor tissues (Figure 7(B)). Our results demonstrated that apoptotic cells observed in the CMX–DOX under OMF treated groups were slightly more obvious than DOX treated group. While the saline control group did not cause significant apoptotic in tumor tissue. Notably, mice after CMX–DOX treatment under OMF were tumor-free and survived for more than 60 days, while the average life spans of mice in the saline control groups were less than 24 days, strongly suggesting CMX–DOX released under OMF to be an efficient agent for *in vivo* treatment of tumors. The results indicate that the CMX–DOX NPs were not noticeably toxic to mice at our tested dose.

## Conclusions

The widely used anticancer agent DOX was successfully conjugated with fluidMAG-CMX *via* an amide bond. FTIR and confocal microscopy were utilized to characterize the whole process. Drug release experiments were conducted to simulate cellular conditions, showing that only 17.3% of the total DOX was cleaved from the conjugates. CMX–DOX showed a triggered-release property when placed in an external magnetic field. There was a significantly increased release of DOX under an OMF compared with DOX release in crude protease, i.e. 70% versus 17.3%. We designed a turn-on and turn-off switch experiment to control drug release and verify the controllable release of CMX–DOX. In *in vivo* experiments, CMX–DOX showed the best anti-tumor effect and lowest toxicity compared with free DOX. This drug delivery system has the potential to become a favored strategy for targeted delivery with reduced side effects.
